# Sustainable strategies to improve indoor comfort and reduce energy consumption in winter: Integration of fly ash and rice husk ash in PCM concrete

**DOI:** 10.1371/journal.pone.0360241

**Published:** 2026-10-15

**Authors:** Mingming Zhang, Qingling Wu, Tingting Lu, Honghao Ying

**Affiliations:** 1 Shaanxi Key Laboratory of safety and durability of concrete structures, Xijing University, Xi’an, China; 2 School of Civil Engineering and Architecture, Wenzhou Polytechnic University, Wenzhou, China; 3 College of Pipeline Engineering, Xi’an Shiyou University, Xi’an, China; Graphic Era Deemed to be University, INDIA

## Abstract

The thermal performance of a building envelope is closely related to its energy consumption. Merging industrial by-products like fly ash (FA) and rice husk ash (RHA) with phase change material (PCM) concrete unlocks a pathway to significantly enhanced thermal insulation properties for envelopes. This study investigated the effects of two different proportions of industrial by-products (5%−15% RHA and 10%−30% FA) on PCM concrete. The test results show that, at the same dosage, RHA is superior to FA in reducing thermal conductivity, especially at an incorporation rate of 15% RHA. Adding 15% RHA and 30% FA resulted in a 2.3 °C increase in the minimum temperature of the PCM concrete. Moderate RHA and FA resulted in a more uniform pore distribution and finer pore size. With increasing RHA content, the capillary pore volume increased and the fractal dimension decreased. In comparison, a higher FA content led to a significant increase in the total pore volume, particularly in the macropore volume, accompanied by a reduction in fractal dimension. The results of the correlation analysis indicated that capillary pores have a significant impact on the thermal properties. It is shown that PCM concrete can be used to obtain available insulated concrete, which has the potential to reduce winter energy consumption.

## 1. Introduction

Currently, limited natural resources are being depleted at an increasing rate. Increased energy consumptions has become a serious reality that people cannot ignore [1–4]. Especially in the construction industry, energy consumption is astonishing [5,6]. To improve indoor thermal comfort during winter, buildings consume large amounts of energy to sustain a comfortable indoor temperature. However, studies have shown that 70% of the heat loss is attributed to the building envelope [7]. Therefore, it is inevitable that the envelope needs to improve its thermal insulation performance, to reduce heat loss.

Improving envelope insulation is an important measure to promote energy efficient buildings [8,9]. In recent years, phase change material (PCM) concrete, as a new type of building material, has been widely concerned by scholars at home and abroad for its potential to improve the thermal insulation performance of envelopes [10–13]. The PCM contained in concrete undergoes phase change within a certain temperature range, absorbing and releasing large amounts of latent heat. Adding PCM to concrete as an envelope can change the heat exchange process between indoors and outdoors. Concrete incorporated with PCM improves thermal comfort indoors in hot regions, reducing the maximum temperature by 3.5 °C [14]. Similarly, in cold regions, the latent heat released by PCM contributes to increasing the minimum indoor temperature when the outdoor ambient temperature decreases, thereby reducing energy consumption related to air conditioning systems [15].

Although PCM incorporated into concrete improves the thermal inertia and alters the heat transfer process. However, the matrix of concrete is still an ordinary matrix [16]. Biomass materials generally have better thermal insulation properties [17,18]. Currently, in power plants of some countries, rice husk is one of the main materials used for biomass power generation [19]. However, the waste produced, known as RHA, can pollute the environment if proper environmental protection measures are not implemented. Therefore, recycling RHA is beneficial for promoting cleaner production [20–22]. RHA is considered a pozzolanic ash active material because it produces large amounts of amorphous silica when incinerated at high temperatures [23,24]. Scholars have added RHA to concrete to improve mechanical and durability properties [25–27]. Unfortunately, there are relatively few research reports on adding RHA into concrete to enhance its thermal insulation performance. The pores formed after incineration of rice husk ash lead to improved thermal insulation performance [28].

Adding RHA to concrete adversely affects its workability, while FA exerts a positive effect on the workability. Therefore, the simultaneous incorporation of RHA-FA into cement will mitigate the adverse effect in flowability caused by the RHA in cement. Despite the extensive study on the application of RHA and FA in concrete, research combining them with PCM concrete lags behind.

This paper aims to systematically investigate the influence of different contents of RHA and FA on PCM concrete. RHA was used to replace cement by 5%, 10%, and 15%, while FA was used to replace cement by 10%, 20%, and 30%. Subsequently, both RHA and FA were integrated into PCM concrete. In the study, the workability of concrete was determined. Then, experimental research was carried out on the compressive strength and splitting tensile strength. Next, the thermal constant were tested. Subsequently, thermal response behavior of concrete specimens within a temperature range of 5 °C to −15 °C was monitored. Using NMR technology, the pore volume and fractal dimension of concrete was analyzed. Finally, the correlation between the pore characteristics of concrete and its macroscopic properties was established through correlation analysis.

## 2. Test materials and research methods

### 2.1 Test materials

RHA was obtained by incinerating rice husks at 600 °C and then milling them in a Los Angeles abrasion tester at 2700 rpm. FA was added to improve the adverse impact of RHA on the flowability of cementitious materials. The ultrafine FA was utilized in this study. The strength grade of the cement used was 42.5 R. The chemical composition of test materials is shown in Table 1. The size distribution of RHA, FA, and cement was determined, shown in Fig 1.

**Table 1:** Chemical composition of the material (wt. %).

	SiO_2_	CaO	Al_2_O_3_	Fe_2_O_3_	MgO	SO_3_	Na_2_O	K_2_O	TiO_2_	MnO	P_2_O_5_
RHA	86.82	1.70	0.47	1.71	0.61	0.55	0.20	5.12	0.05	0.45	1.89
FA	43.46	10.05	30.57	9.12	2.08	0.53	1.02	0.67	0.55	0.05	1.30
Cement	18.68	64.27	4.01	3.97	2.87	3.26	0.12	0.43	0.57	0.10	1.05

**Fig 1:**
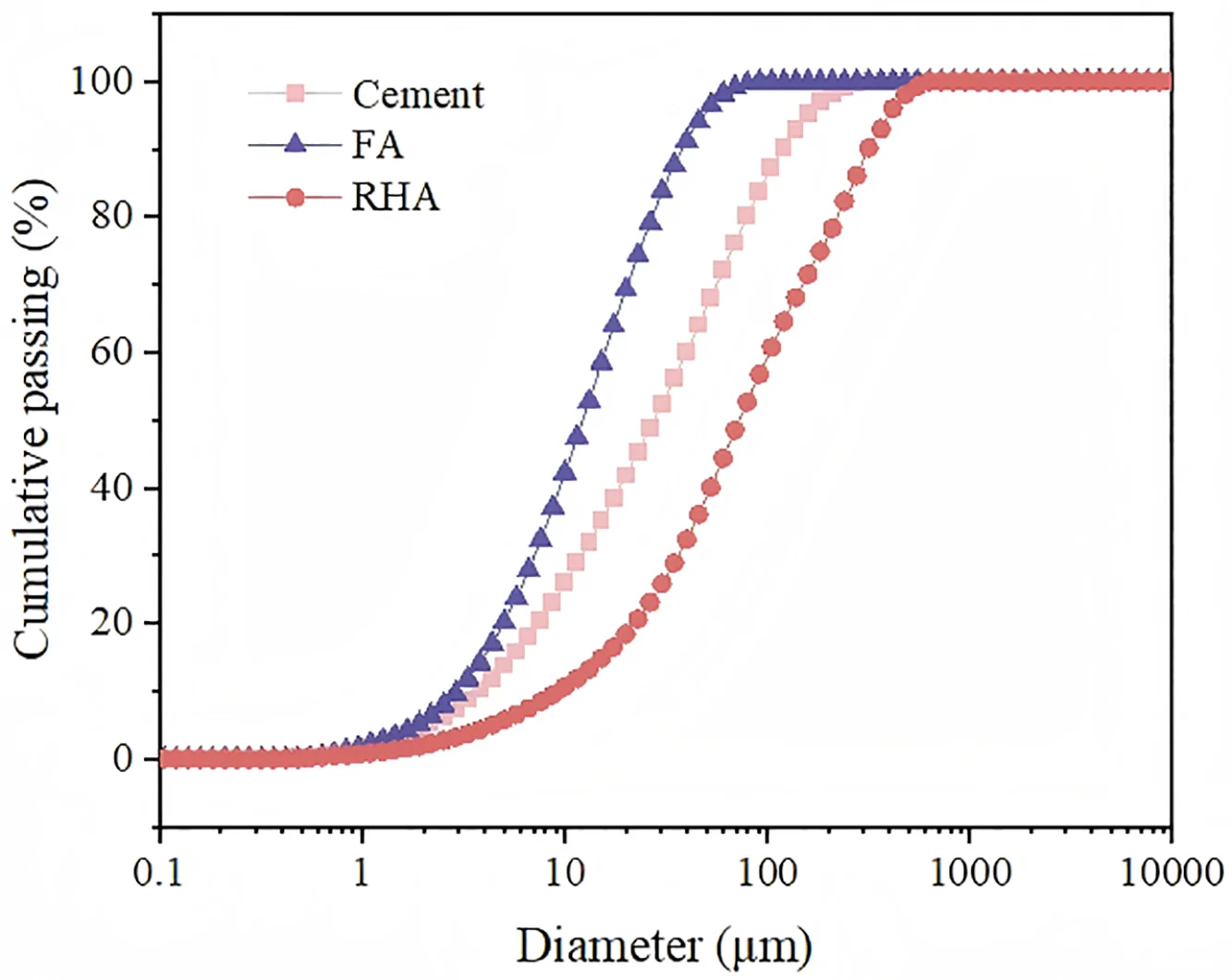
Particle size distribution.

The coarse aggregate in concrete in this study was PCM aggregate, with a particle size ranging from 5 to 20 mm, shown in the Fig 2. The PCM aggregate used in this study was uniformly mixed with three types of aggregates (dodecane, tridecane, and tetradecane) as raw materials at a mass ratio of 4:3:3. The specific preparation method of the PCM aggregate can refer to relevant research papers [29]. The basic physical properties of dodecane, tridecane, and tetradecane are shown in Table 2. Since the densities of the three PCMs are similar, the proportion of the PCM in the aggregate is essentially the same, at approximately 10%.

**Table 2:** Thermal performance of PCMs.

PCM	Dodecane	Tridecane	Tetradecane
Density (g/ml)	0.7487	0.7560	0.7628
Phase change temperature (℃)	−12.86	−7.84	4.26
Latent heat (J/g)	200.5	142.9	206.3

**Fig 2:**
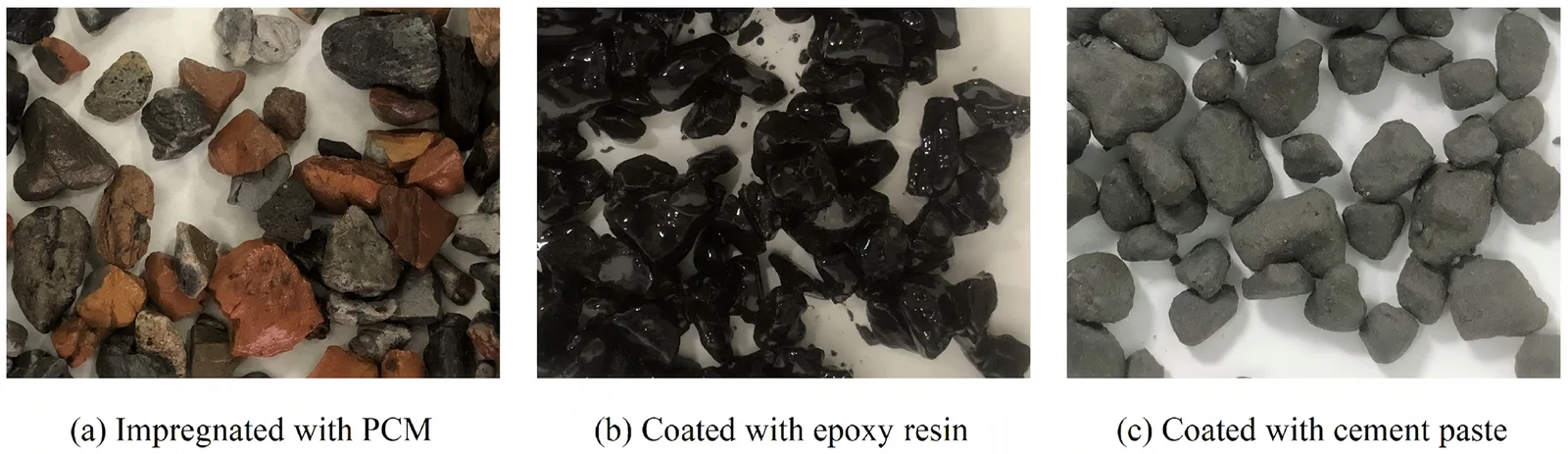
PCM aggregate. (a) Impregnated with PCM; (b) Coated with epoxy resin; (c) Coated with cement paste.

The mixture used to prepare the concrete samples is shown in Table 3. RHA and FA were used as supplementary cementitious materials to replace cement, forming sustainable RHA-FA-cementitious materials. In this study, RHA replaced cement at 5%, 10%, and 15% by mass, while FA replaced cement at 10%, 20%, and 30% by mass.

**Table 3:** Mixture of concrete (kg/m^3^).

Mixture	RHA content	FA content	Cement	RHA	FA	Sand	Gravel	PCM aggregate	Water	SP
C0	0%	0%	550	0	0	652	1080	0	176	5.5
A0	0%	0%	550	0	0	652	0	560	176	5.5
A1	5%	10%	467.5	27.5	55	652	0	560	176	5.5
A2	10%	10%	440	55	55	652	0	560	176	5.5
A3	15%	10%	412.5	82.5	55	652	0	560	176	5.5
A4	5%	20%	412.5	27.5	110	652	0	560	176	5.5
A5	10%	20%	385	55	110	652	0	560	176	5.5
A6	15%	20%	357.5	82.5	110	652	0	560	176	5.5
A7	5%	30%	357.5	27.5	165	652	0	560	176	5.5
A8	10%	30%	330	55	165	652	0	560	176	5.5
A9	15%	30%	302.5	82.5	165	652	0	560	176	5.5

### 2.2 Research methods

#### 2.2.1 Workability test.

The required testing instrument is the slump cone, which is open at both ends, with a base diameter of 200 mm and a top diameter of 100 mm. The concrete was vibrated and compacted each time it was poured. Then, it was lifted vertically when the cone was filled. When the concrete ceased slumping or reached 30 seconds, the variance in height was recorded as the slump. When the concrete stopped flowing or reached 50 seconds, taking the average vertical diameter as the test slump flow value. The value was precise within 1 mm, and the outcome was rounded to 5 mm.

#### 2.2.2 Strength test.

After curing for 7 and 28 days, the strength was measured. A 2000 kN electro-hydraulic servo universal testing machine was employed for the tests. The loading rate was set at 3 mm/min for the compressive strength test and 1.5 mm/min for the tensile strength test [30]. The specimens were prepared with dimensions of 100 mm × 100 mm × 100 mm. All tests were performed in three times, and the average values were adopted as the final strength results.

#### 2.2.3 HotDisk test.

The HotDisk thermal constant analyzer was used to measure thermal constants. When current passes through the sensor of the analyzer, the heat contributed to an increase in the probe’s temperature, which was determined by the change in the electric resistance of the probe. The instrument determined the thermal constant of the material by analyzing the time-dependent temperature rise recorded during the test, followed by a fitting procedure [31,32]. Before the test, both faces of the concrete were ground smooth to improve the fit of the probe, which was then placed in the middle of the two concrete samples. The whole test procedure was conducted at room temperature.

#### 2.2.4 Thermal response Tests.

Experiments were conducted using temperature sensors and a temperature test chamber, and temperature curves were obtained. The temperature sensor was embedded at the center of the specimen to measure the internal temperature changes. The temperature setting of the temperature test chamber was −15 °C to 5 °C, indicating that the ambient temperature varied within this range. The temperature is recorded through a temperature recorder, with a temperature monitoring frequency of 60 seconds.

#### 2.2.5 NMR test.

Nuclear Magnetic Resonance (NMR) technology utilizes the spin signal properties generated by the water or hydrogen atoms in concrete in a magnetic field to identify pore size and distribution, which can be expressed as Formula (1) [33]. *T*_2_ is the transverse relaxation time, *S*/*V* is the pore surface area-volume ratio, and *ρ*_2, sur_ is the surface relaxivity. The concrete cubic specimens were vacuum-saturated with water for 24 h before the test to fill the interior pores with water. Then, the specimens were wrapped in a non-magnetic film to prevent water evaporation.


$$\begin{array}{c} \frac{\text{1}}{T_{\text{2}}}\text{=}\rho_{2,sur}\left(\frac{S}{V}\right) \end{array}$$ (1)

## 3. Results and discussion

### 3.1 Workability

Fig 3 illustrates the workability performance of different concrete mixture. The slumps of concrete C0 and concrete A0 were 125 mm and 70 mm, respectively. PCM aggregate had an adverse effect on workability, Rahemipoor et al. integrated the macro-encapsulated PCM into geopolymer cement, which also decreased the workability [10]. The addition of 5% RHA and 10% FA to A0 concrete increased the slump to 190 mm, and the concrete flowability was improved with a slump flow of 525 mm. Therefore, acceptable workability can be obtained under the ternary cementitious system.

**Fig 3:**
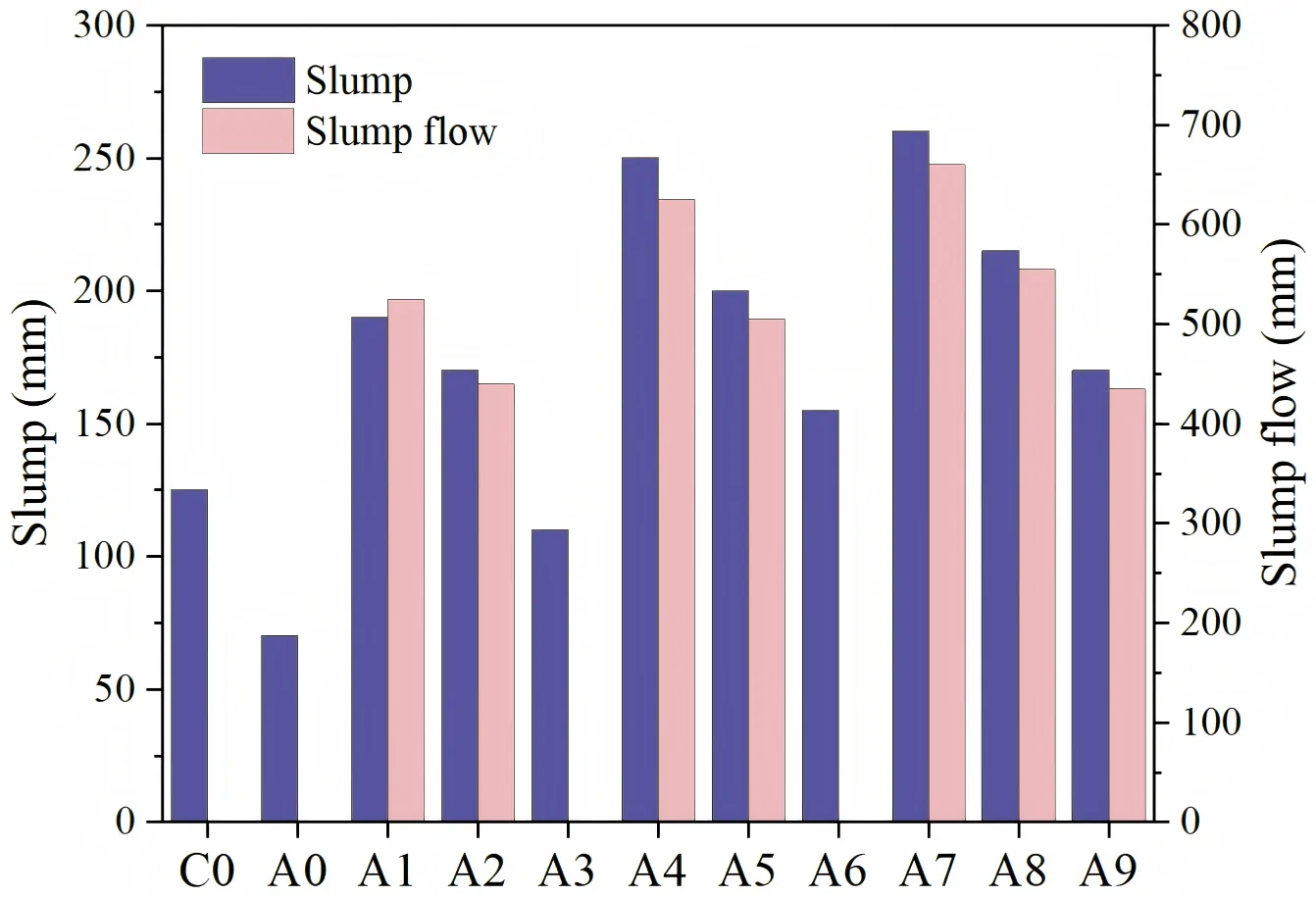
Slump and slump flow of concrete.

With increasing RHA content, concrete workability decreased significantly. The slump decreased by 34.62% to 42.11% with a 15% RHA addition compared to a 5% RHA addition. Due to high specific surface area and internal pores, more water can be absorbed by RHA, and reducing the content of free water after being incorporated into concrete. In addition, due to the irregular shape, the frictional resistance may increase, leading to agglomeration of RHA particles, thereby reducing the workability of concrete [34].

However, with FA increased, the slump increased. Similar results were reported by Farahani et al. [35]. Moreover, a higher amount of FA altered the consistency of concrete, causing it to start flowing, as compared to A3, A6, and A9. With the FA content increased to 30%, the slump improved by 26.47% to 54.54%. In 5% RHA-30% FA concrete (A7), the slump and slump flow reached 260 mm and 660 mm, respectively, demonstrating high flowability performance. This can be attributed to the fact that the spherical particle shape of FA reduces friction and collision with other micro-particles, thereby decreasing the yield stress of the concrete and improving its workability.

### 3.2 Mechanical properties

Fig 4 shows the compressive strength of different concretes cured for 7 and 28 d. The strength of the PCM concrete containing 5% RHA and 10% FA (A1) increased by 10.60% and 12.71% at 7 and 28 days, respectively, compared to the reference concrete. This enhancement can be attributed to two main factors. First, the differences in particle size among these particles contribute to an increased bulk density of the cementitious matrix, leading to enhanced strength. Furthermore, the pozzolanic reactions of RHA and FA with the hydration products result in secondary hydration, which further fills the pores and densifies the microstructure, thereby improving the concrete strength [36,37].

**Fig 4:**
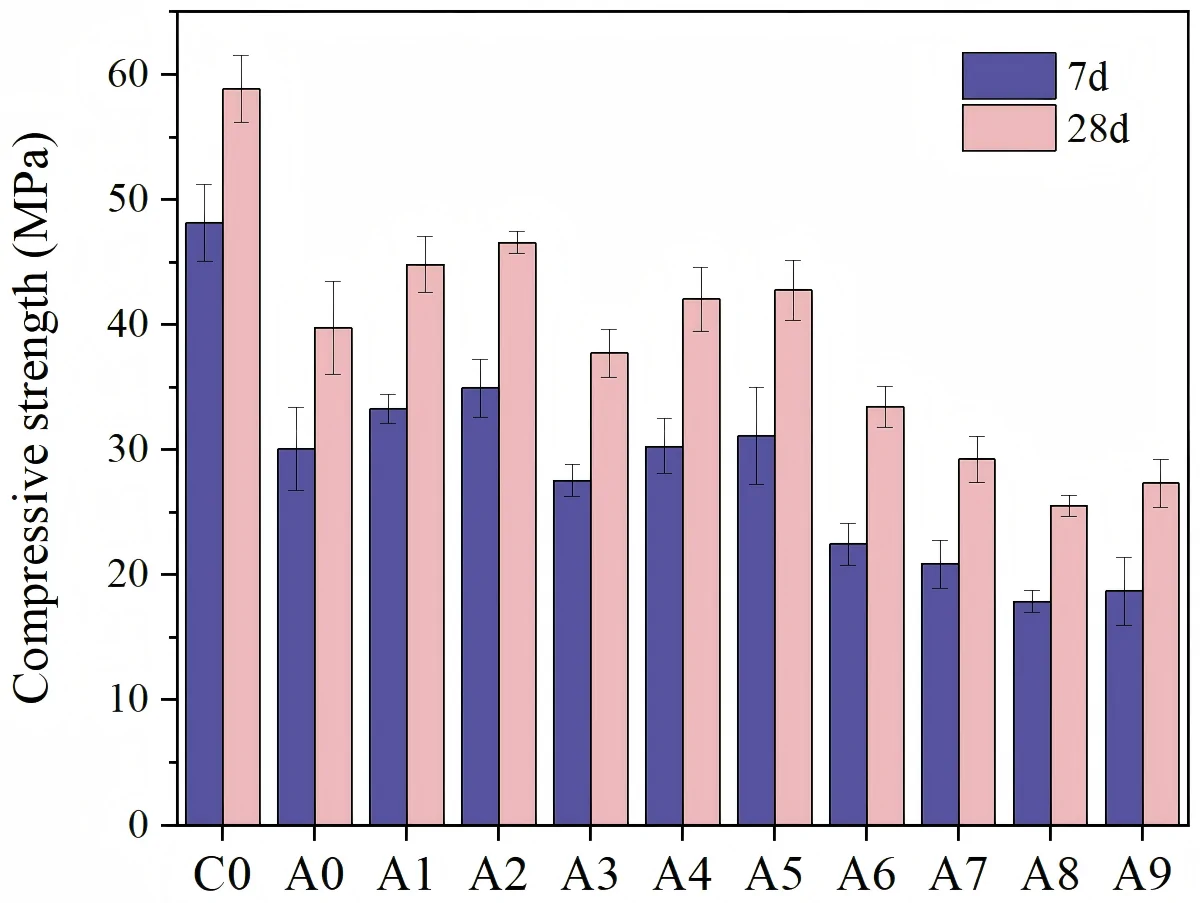
Compressive strength of concrete.

An appropriate amount of RHA enhanced concrete strength [38]. In most mixtures (groups A1-A3 and A4-A6), strength initially increased and then decreased with higher RHA content. For example, within the A1-A3 group, the 28-day strength of concrete with 10% RHA (A2) was 3.97% higher than that with 5% RHA (A1). However, when the RHA content was further increased to 15% (A3), the strength decreased by 15.76%. Notably, a different trend was observed in the A7-A9 group, where strength consistently decreased as the RHA content increased from 5% to 15%. These results demonstrate that while an optimal RHA dosage improves strength, an excessive amount leads to a strength loss.

The content of FA also significantly influences concrete strength. For a given RHA level, mixes were prepared with three FA contents: 10% (groups A1, A4, A7), 20% (groups A2, A5, A8), and 30% (groups A3, A6, A9). Across these series, the corresponding strength decreased progressively with higher FA content. Specifically, increasing FA from 10% to 20% resulted in strength reductions ranging from 9.04% to 18.52%. A further increase to 30% FA led to more substantial decreases of 32.13% to 48.88%. When cement is partially replaced, the hydration products produced by the pozzolanic effect are beneficial to enhancing mechanical properties. When the replacement amount exceeds the optimal replacement amount, the decrease in cement content results in inadequate hydration products. This leads to a lack of calcium hydroxide used for the reaction, resulting in lower hydration levels. Meanwhile, the significant replacement of cement also leads to a decrease in strength.

Fig 5 shows the splitting tensile strength of concrete. Adding 10% RHA was most beneficial to the splitting tensile strength of concrete. For example, the strength of concrete A5 (containing 10% RHA) is 1.99% and 13.97% higher than that of concrete containing 5% and 15% RHA, respectively. The addition of an appropriate amount of FA increased the splitting tensile strength of concrete. The strength of mixture with 10% FA (e.g., A2) was higher than concrete with 20% and 30% FA by 3.34% and 23.67%, respectively. The mixture containing 10% RHA and 10% FA showed the highest splitting tensile strength, which was 15.93% higher than reference concrete.

**Fig 5:**
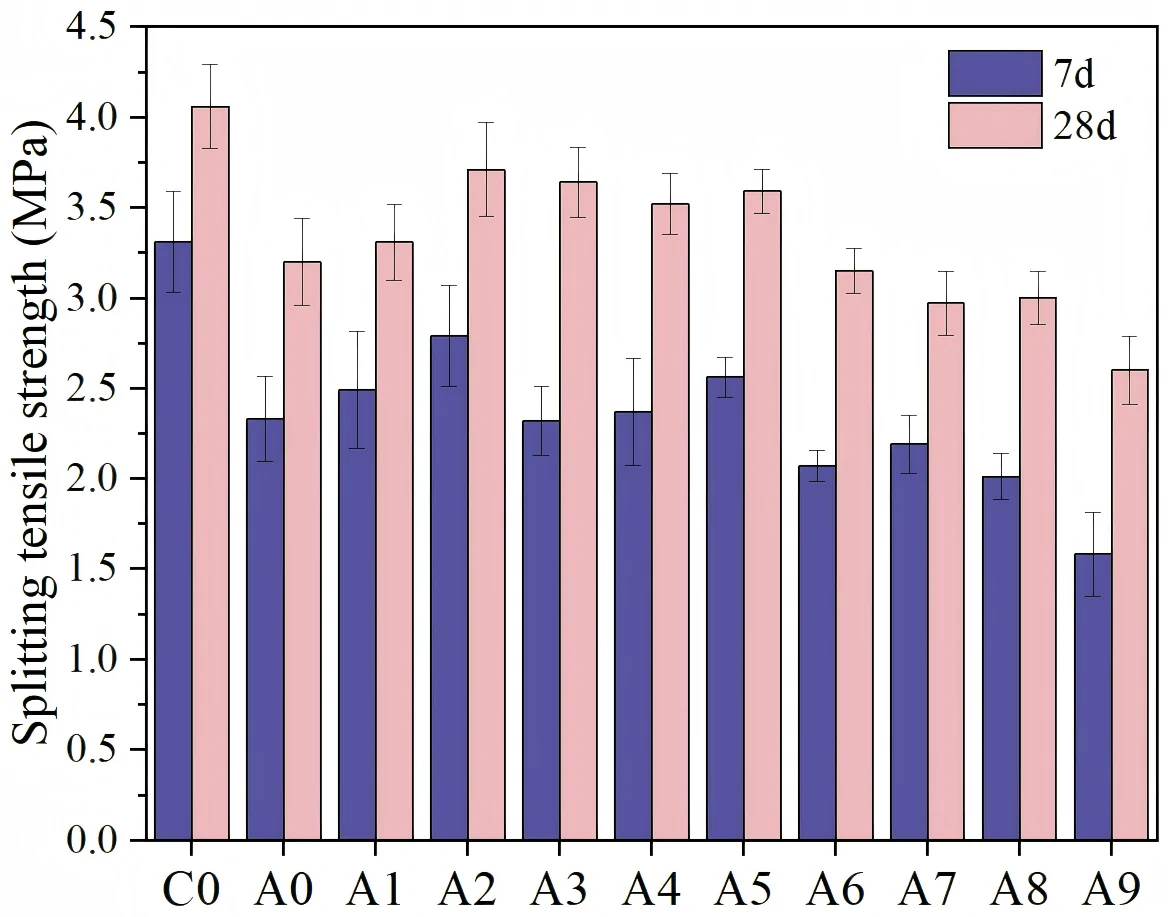
Splitting tensile strength of concrete.

### 3.3 Thermal constants

Fig 6 illustrates the constants of various mixtures. Upon incorporating RHA and FA into PCM concrete, both the thermal conductivity and thermal diffusivity decreased. Notably, the concrete containing 5% RHA and 10% FA (A1) exhibited the minimal change relative to concrete A0, with a mere 0.92% decrease in thermal conductivity and a 2.06% decrease in thermal diffusivity. This minor influence can be attributed to the balance between two mechanisms: on one hand, the inherent porosity of RHA tends to increase the overall pore volume and reduce the thermal conductivity; on the other hand, the fine particles of RHA and FA also enhance the particle bulk density when combined with cement. The increase in bulk density reduces interfacial pores, making the microstructure denser, thereby enhancing heat transfer. The combined effect of these two factors is that the impact of low-content (5%) RHA on overall thermal performance is negligible.

**Fig 6:**
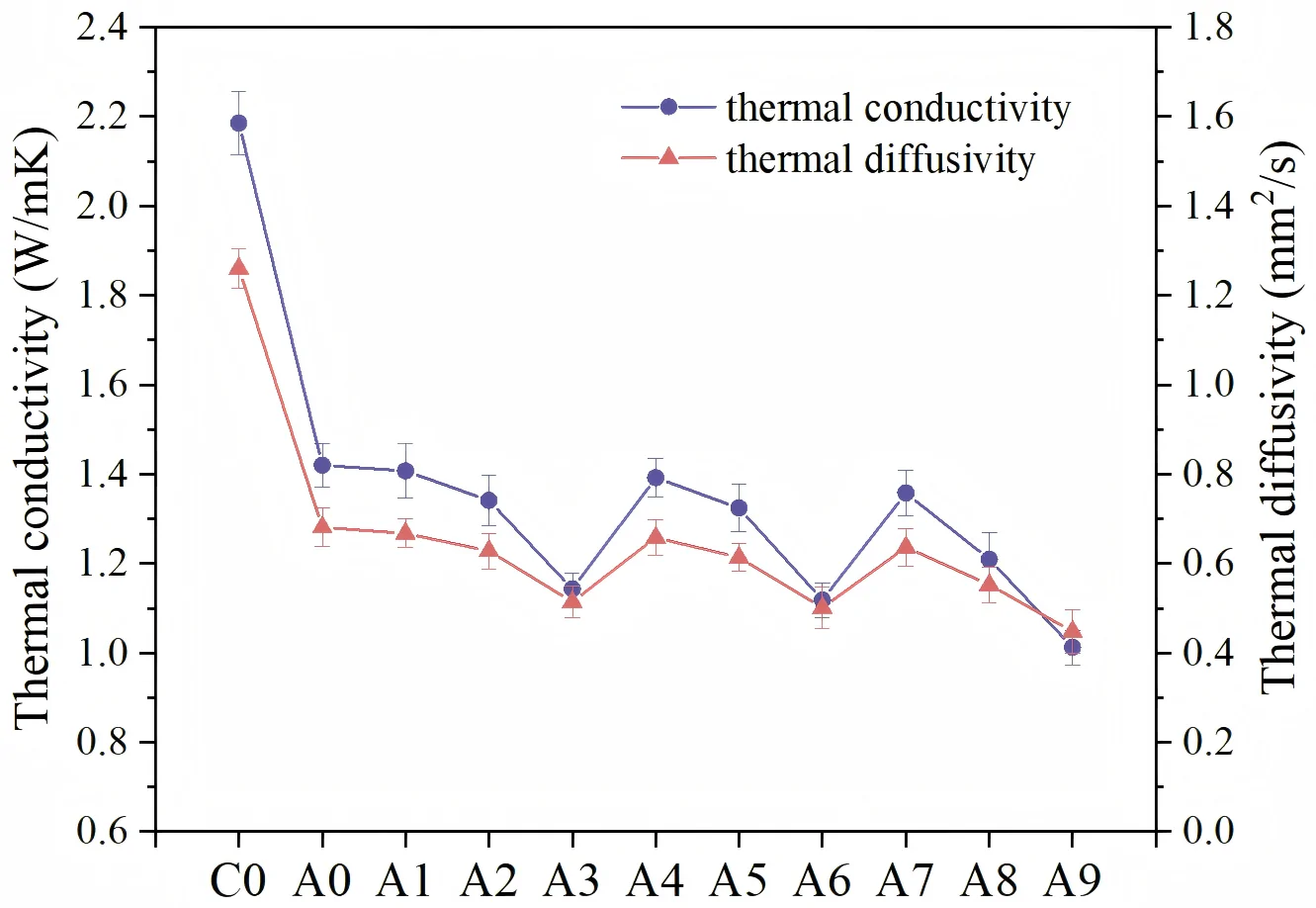
Thermal performance of concrete.

The thermal constants of concrete exhibited a monotonic decline with increasing RHA content. Specifically, compared to 5% RHA, concrete with 15% RHA exhibited reductions of approximately 18–25% in conductivity and 23–29% in diffusivity. This significant decrease is primarily attributed to the increased porosity introduced by RHA, which impedes heat transfer through the material matrix [39]. In addition, the predominantly micrometer-scale pores within RHA and its homogeneous distribution in the matrix may also contribute to the reduction in thermal constants. The increase in the FA content had little effect on the thermal constants. Concrete with 30% FA showed only a 3% to 11% decrease in conductivity and a 5% to 12% decrease in diffusivity compared to reference concrete.

### 3.4 Time-temperature curve

Fig 7 illustrates time-temperature test curves. As the ambient temperature dropped, the ordinary concrete (C0) exhibited the fastest cooling rate, whereas all other mixtures cooled more gradually, resulting in a significant delay in reaching their minimum temperatures. While the ordinary concrete (C0) cooled to −14.4 °C, the PCM concrete (A0) maintained a significantly higher temperature of −11.5 °C. Furthermore, all RHA-FA modified concretes exhibited even better thermal inertia performance, with temperatures remaining above −11 °C. Notably, the mixture containing 15% RHA and 30% FA (A9) showed the least temperature drop, reaching only −8.9 °C. The study by Chin et al. also demonstrated that incorporating PCM aggregates enhanced the thermal damping effect, thereby attenuating temperature fluctuations [40]. In this study, RHA and FA incorporated into PCM concrete can further decrease the rate of temperature change. The peak temperature of concrete containing 15% RHA and 30% FA (A9) was delayed by approximately 19 min as compared to PCM concrete (A0) and by 43 min as compared to ordinary concrete (C0). The tests confirm that RHA effectively reduces the peak temperature and delays its occurrence. When the ambient temperature began to rise, concrete mixed with RHA and FA exhibited a pronounced thermal lag phenomenon, where the temperature does not immediately increase. This behavior indicates that the addition of these materials enhances the thermal damping capacity of concrete, slowing down its response to ambient temperature changes. Janga et al. [41] also reached a similar conclusion.

**Fig 7:**
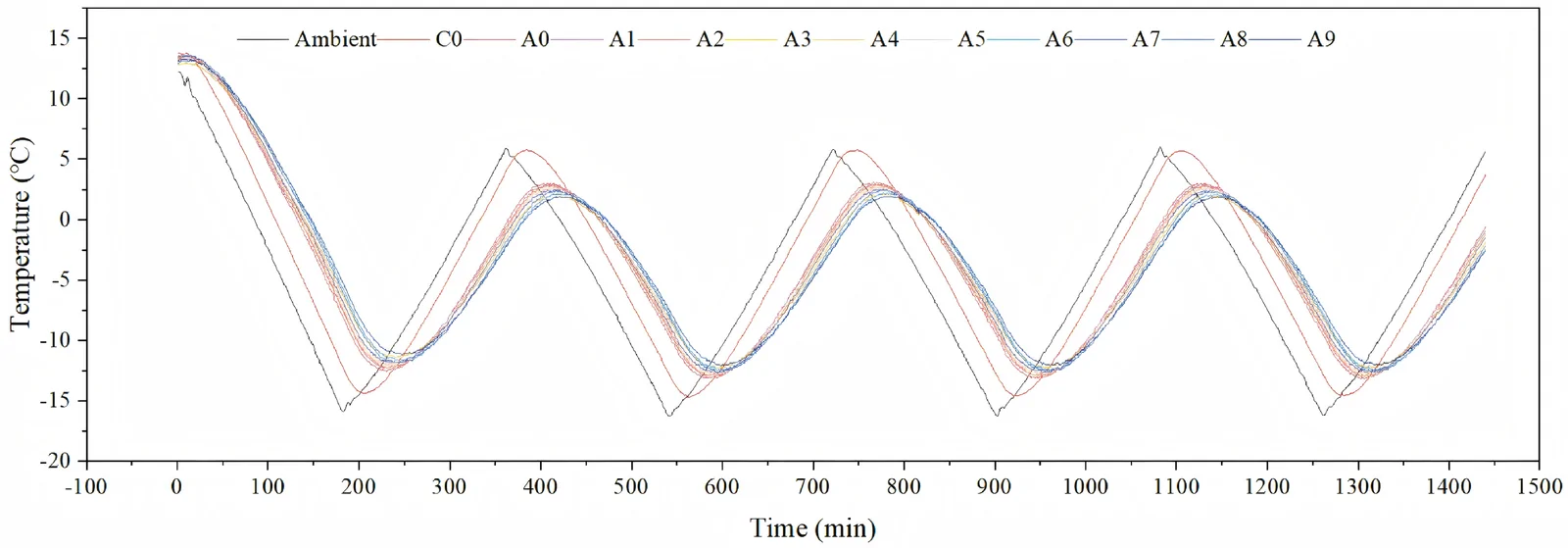
Time-Temperature curve of concrete.

Fig 8 compares the thermal response of concrete across different incorporation levels of RHA and FA. All RHA-FA mixed concrete mixtures to maintain smaller temperature fluctuations than the reference concrete (A0). Among them, the mixture with 15% RHA and 30% FA (A9) exhibited the largest temperature difference, reaching 2.3 °C compared to concrete A0. A significant difference of 1.4 °C was also observed for the mixture containing 15% RHA and 10% FA (A3).

**Fig 8:**
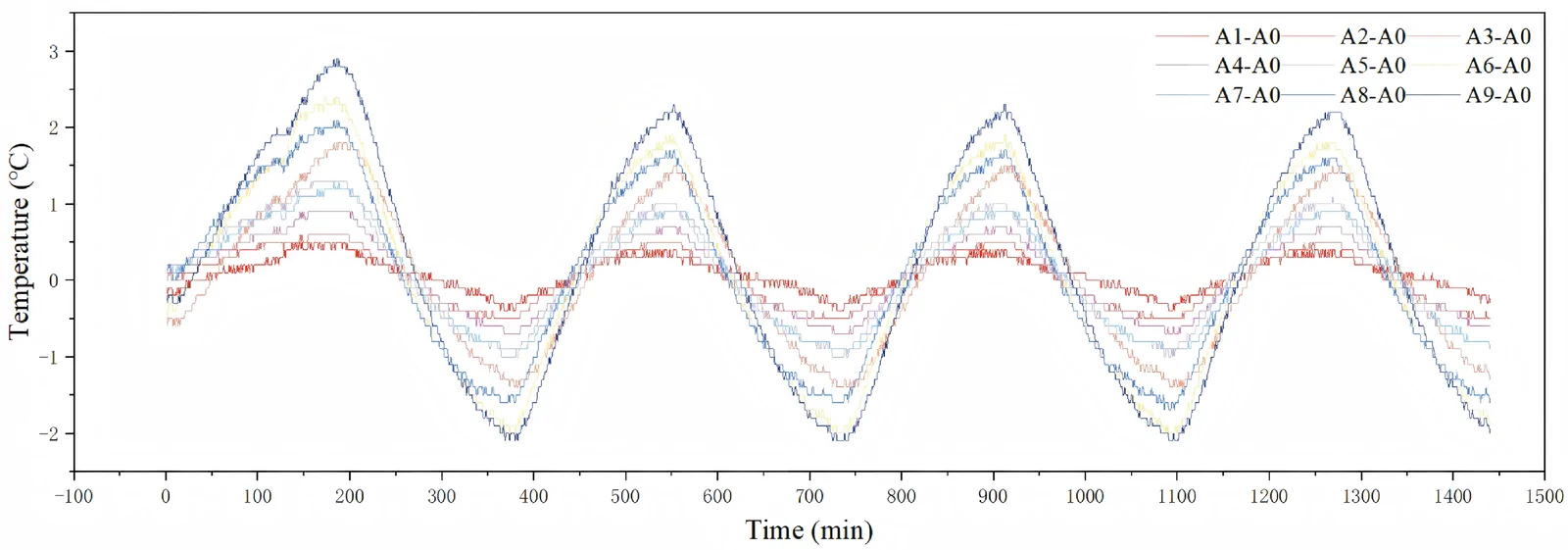
Temperature difference between RHA-FA concrete and PCM aggregate concrete.

Concrete A3 (containing 15% RHA, 10% FA) and concrete A4 (containing 5% RHA, 20% FA) had the same total replacement level. However, the temperature difference for A4 was significantly lower (0.7 °C) than that for A3 (1.4 °C). A similar trend was observed for another group, concrete A6 (containing 15% RHA, 20% FA) and concrete A7 (containing 5% RHA, 30% FA), where the mixture with the higher RHA content again performed better. These results indicate that, at an equivalent total replacement level, RHA is more effective than FA in improving the thermal inertia of concrete.

### 3.5 NMR

Fig 9a presents the T_2_ relaxation time spectra for RHA-FA PCM concrete and reference concrete. In the T_2_ relaxation spectrum, the signal amplitude (vertical axis) reflects the volume of pores corresponding to a given relaxation time, while the transverse relaxation time T_2_ (horizontal axis) determines the pore size. Based on the T_2_ relaxation time, pores are typically classified into three categories: micropores, capillary pores, and macropores, the pore sizes are respectively ≤100 nm, 100–1000 nm, and >1000 nm, with relaxation times respectively ≤ 2.5 ms, 2.5 ms–25 ms, and >25 ms [29,42,43]. Small pores (less than 100 nm) have little effect on concrete strength, while pores larger than 100 nm will adversely affect concrete strength [44].

**Fig 9:**
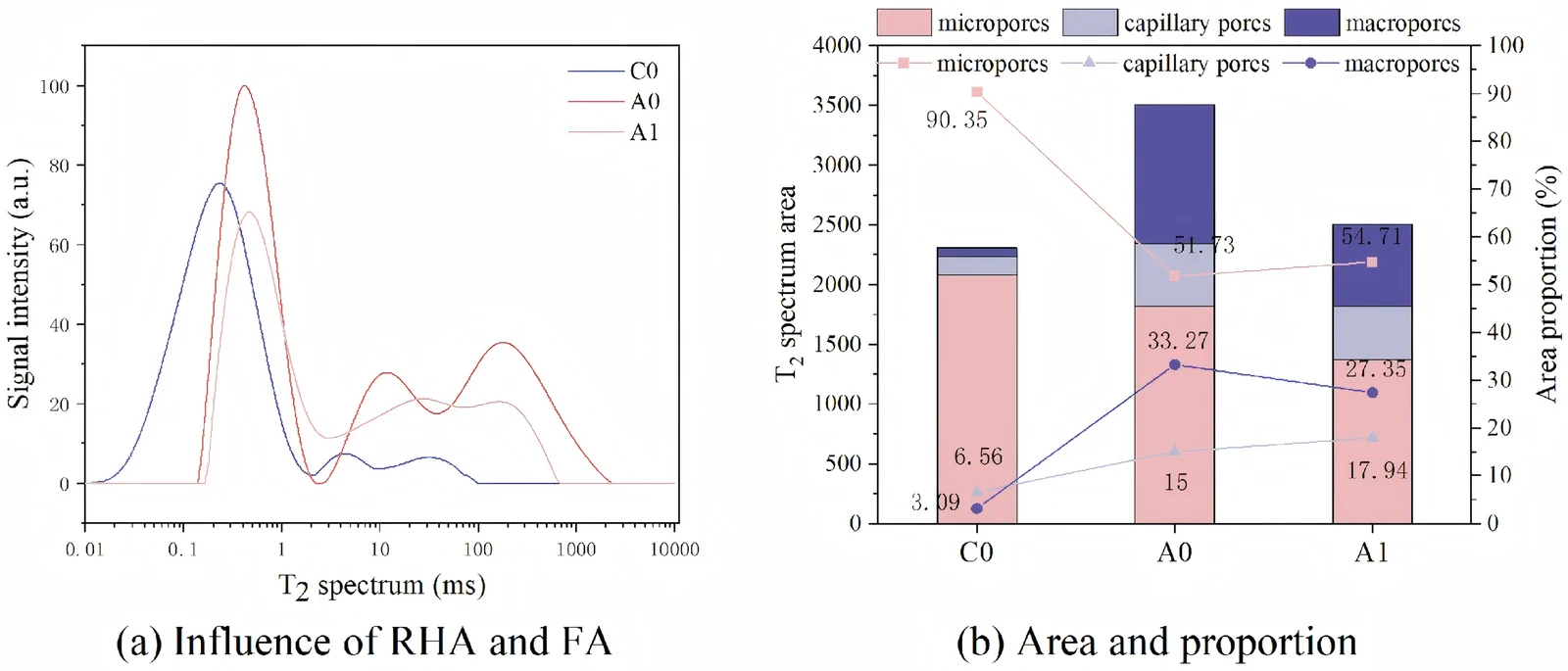
T_2_ spectrum of concrete. (a) Influence of RHA and FA; (b) Area and proportion.

A rightward shift was observed in the T_2_ spectrum when comparing PCM concrete (A0) to ordinary concrete (C0), and the signal intensity increased, indicating that the pore size and volume of the concrete increased. Concrete A1 (containing 5% RHA-10% FA) had a smaller pore size range and a smoother curve. The physical filling and pozzolanic effects of RHA and FA contributed to pore structure refinement, thereby promoting a more homogeneous pore distribution and a decrease in total porosity [45–47]. Fig 9b compares the total pore volume and the pore size distribution. The incorporation of PCM aggregate increased the total pore volume. Specifically, in PCM aggregate concrete (A0), the pore volume was 1.5 times greater than that of concrete C0. The rise of volume in capillary pores and macropores was evident, and these increased pore volumes can negatively impact the strength. However, the subsequent addition of RHA and FA effectively counteracted this effect, reducing the total pore volume by 28.58%. Notably, the volume of macropores was reduced by 41.28%, a key factor contributing to the enhancement of concrete strength.

Fig 10a compares the pore structure of concrete with different RHA addition levels (5%, 10%, and 15%). The signal intensity corresponding to capillary pores increased progressively with higher RHA content. This trend can be attributed to the inherent microporosity of RHA particles themselves, whose increasing volume contributes directly to the capillary pore volume within the composite. In contrast, the signal intensity for macropores decreased initially but then increased at the highest RHA dosage. This indicates that a moderate amount of RHA effectively refines the pore structure through filler and pozzolanic effects, reducing macropore volume. However, an excessive addition may introduce additional voids or disrupt accumulation, leading to a resurgence of macropores. Consequently, the use of an appropriate amount of RHA in concrete may contribute to a narrower and more uniform pore size distribution, which in turn may promote strength development.

**Fig 10:**
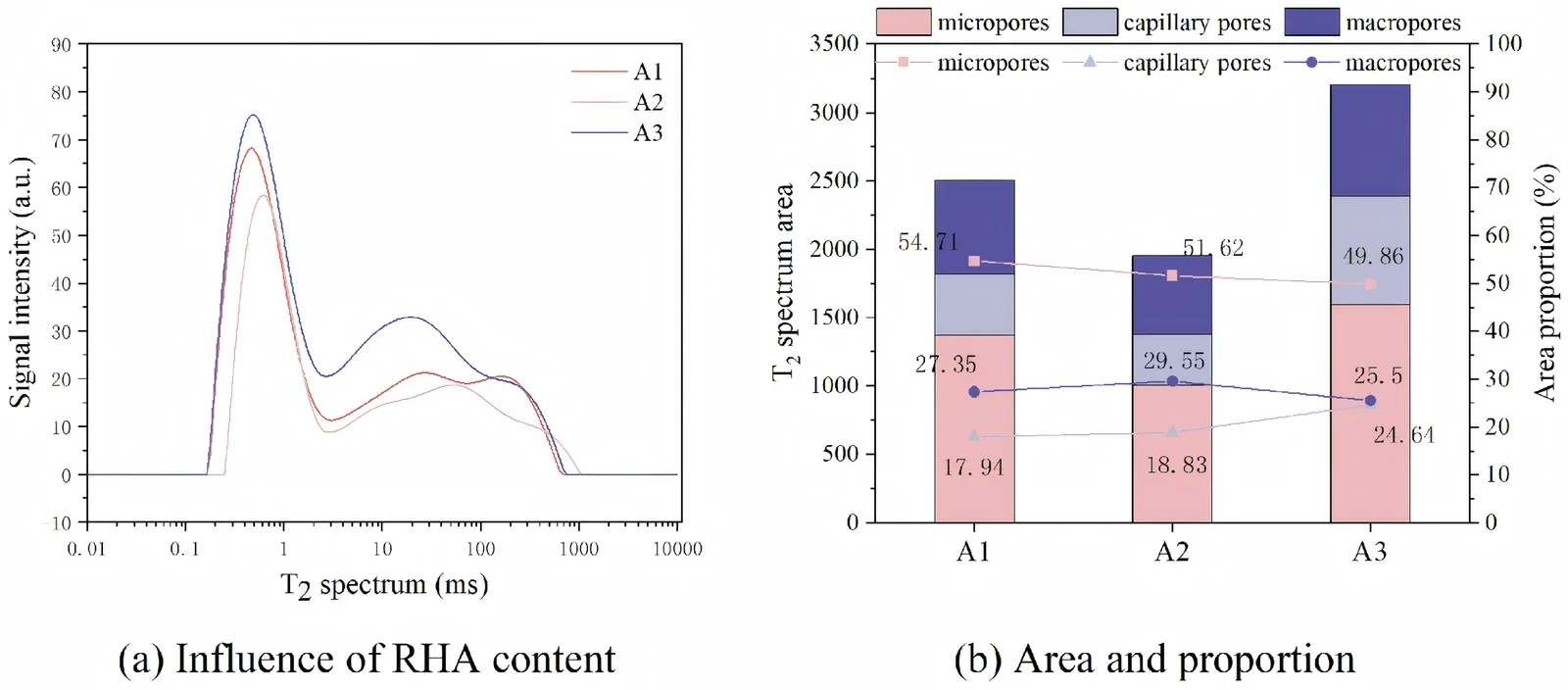
T_2_ spectrum of concrete. (a) Influence of RHA content; (b) Area and proportion.

Fig 10b compares the pore characteristic proportion of concrete with different RHA contents. The total pore volume decreased by 21.95% with a 10% RHA addition (A2) compared to a 5% RHA addition (A1). Although the reduction in total pore volume increased the thermal conductivity, adding more RHA increased micron-sized uniformly distributed pores. The uniform dispersion of RHA in the cementitious system facilitated the reduction of thermal conductivity, resulting in lower thermal conductivity in the concrete with 10% RHA (A2) compared to that with 5% RHA (A1). Concrete with 15% RHA (A3) showed a 27.89% increase in porosity compared to concrete with 5% RHA (A1), which adversely affects the strength.

Fig 11a presents the evolution of the pore structure with increasing FA content (10%, 20%, and 30%). The T_2_ spectrum reveal that the volume of all pore types increased with higher FA dosage, with the signal intensities for micropores and macropores showing the most pronounced rise. The significant expansion of macropore volume is particularly detrimental to concrete strength, a finding consistent with the mechanical test results in Section 3.2. Fig 11b quantifies these changes in total porosity and pore size distribution. Compared to 10% FA, the total pore volume increased by 7.16% and 36.73% for mixtures containing 20% and 30% FA, respectively. More critically, the volume of macropores increased by 18.61% and 53.76%, respectively. This substantial increase in porosity, especially in the harmful macroporous range, is due to the excessive replacement of cement by FA. At high replacement levels, the reduced cement content leads to insufficient hydration, while the pozzolanic reaction of the abundant FA is limited, collectively resulting in a more porous microstructure.

**Fig 11:**
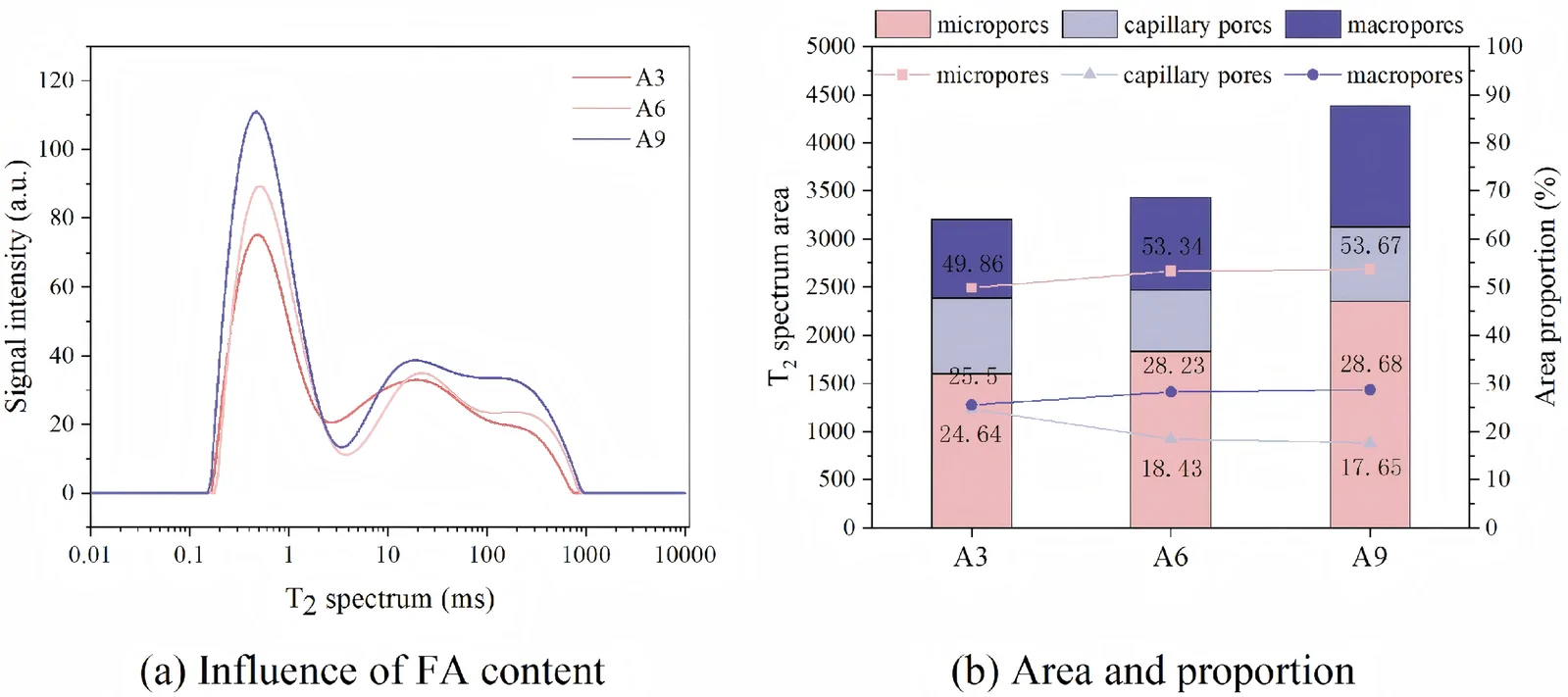
T_2_ spectrum of concrete. (a) Influence of FA content; (b) Area and proportion.

In summary, the pore structure can be effectively refined by the incorporation of optimal dosages of RHA and FA. This refinement results from the combined physical filling and pozzolanic effects, leading to a densified microstructure. The pore structure is filled with fine particles and more products are generated from the pozzolanic reaction. Conversely, excessive dosages of RHA and FA can disrupt the hydration process, resulting in a net increase in porosity and coarsening of the pore structure.

### 3.6 Fractal dimension

Nuclear magnetic resonance (NMR) technology provides a powerful means for non-destructively characterizing the pore size distribution within concrete. However, a comprehensive assessment of the macroscopic properties of concrete requires consideration beyond mere pore distribution, the fractal dimension, is also a critical factor. The fractal dimensions corresponding to different pore size ranges can be calculated using Formula (2).


$$\begin{array}{c} lgS_{v}=(3-D)lgT_{\text{2}}+(D-3)lgT_{2max} \end{array}$$ (2)

Wherein, T_2_ is the transverse relaxation time, T_2max_ is the maximum transverse relaxation time, S_v_ is the proportion of pore volume within the corresponding relaxation time range, and D is the fractal dimension.

Fig 12 presents the lgS_v_–lgT_2_ curves of concrete C0, A0, and A1, while Table 4 lists the corresponding fractal dimensions derived from fitting these curves. The fractal dimensions of capillary pores and macropores in all concrete specimens exhibited distinct fractal characteristics, which can be used to characterize the complexity of pore. After the complete replacement of coarse aggregates with PCM aggregates, the fractal dimensions of capillary pores and macropores in concrete decreased by 0.0921 and 0.0712, respectively. The water absorption of the cementitious shell surrounding PCM aggregates increases the local water-to-cement ratio upon mixing, resulting in a less complex pore structure. Incorporating 5% RHA and 10% FA altered this trend: the fractal dimension of capillary pores remained largely unchanged, while that of macropores increased by 0.0246. This indicates that a suitable dosage of RHA and FA can enhance the complexity of the pore structure.

**Table 4:** The fractal dimension of different concrete.

	D_micro_	K	R^2^	D_capillary_	K	R^2^	D_macro_	K	R^2^
C0	0.6166	0.00%	0.7178	2.97	0.00%	0.9861	2.9976	0.00%	0.3433
A0	1.6071	160.64%	0.6860	2.8779	−3.10%	0.9630	2.9264	−2.38%	0.8637
A1	1.5252	147.36%	0.6913	2.8754	−3.19%	0.9956	2.951	−1.55%	0.7553

Wherein, K represents the relative growth rate.

**Fig 12:**
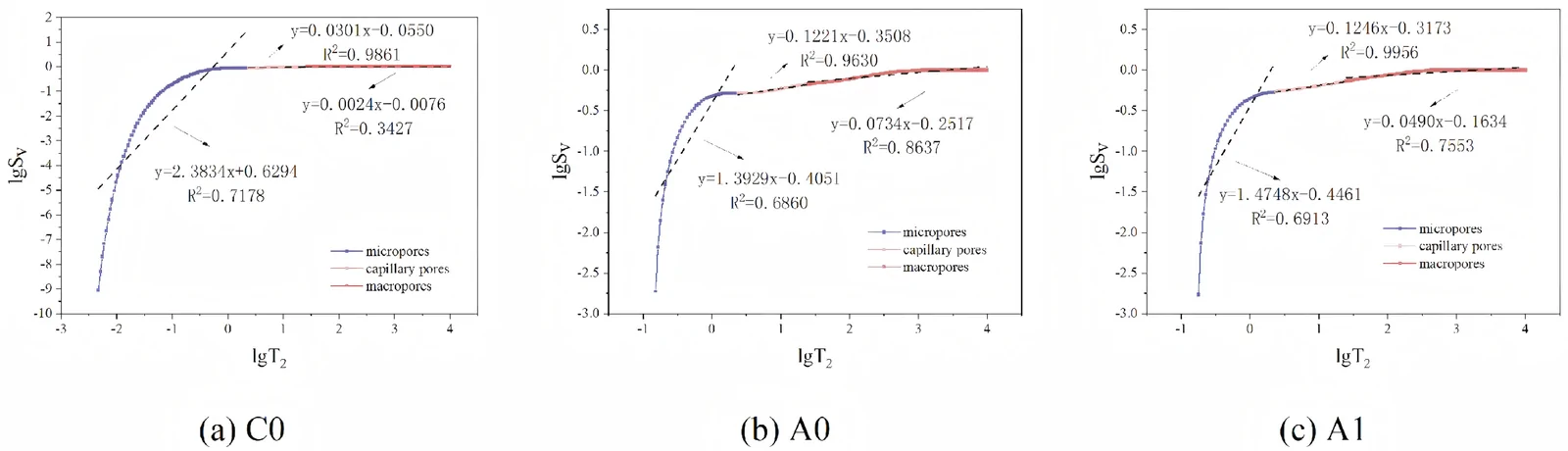
lgT_2_-lgS_v_ curve of different concrete. (a) C0; (b) A0; (c) A1.

Fig 13 and Table 5 show the fractal dimensions of PCM concrete incorporating 5% to 15% RHA, respectively. With the increase in the replacement level of RHA, the fractal dimension of capillary pores decreased continuously. Compared with the concrete mixed with 5% RHA, the fractal dimension of capillary pores in the concrete with 15% RHA decreased by 0.0551. This decline can be attributed to the intrinsic microporosity of RHA particles and the free water they retain, which offsets the pore-filling benefits of RHA’s pozzolanic reaction, thereby reducing the overall complexity of the capillary pore network. In contrast, the fractal dimension of macropores remained relatively stable across different RHA dosages. This stability may be explained by two factors: first, the improved workability contributed by FA helps maintain a uniform mixture, preventing the coarsening of the macroporous structure; second, the products from RHA’s pozzolanic reaction may partially fill existing macropores, counteracting any tendency for complexity to decrease.

**Table 5:** The fractal dimension of concrete with different RHA contents.

	D_micro_	K	R^2^	D_capillary_	K	R^2^	D_macro_	K	R^2^
A1	1.5252	−147.36%	0.6913	2.8754	3.19%	0.9956	2.951	1.55%	0.7553
A2	1.3594	15.41%	0.7141	2.8614	0.57%	0.9966	2.9471	−0.71%	0.7776
A3	1.5371	4.36%	0.7167	2.8203	2.00%	0.9993	2.9576	−1.07%	0.7467

**Fig 13:**
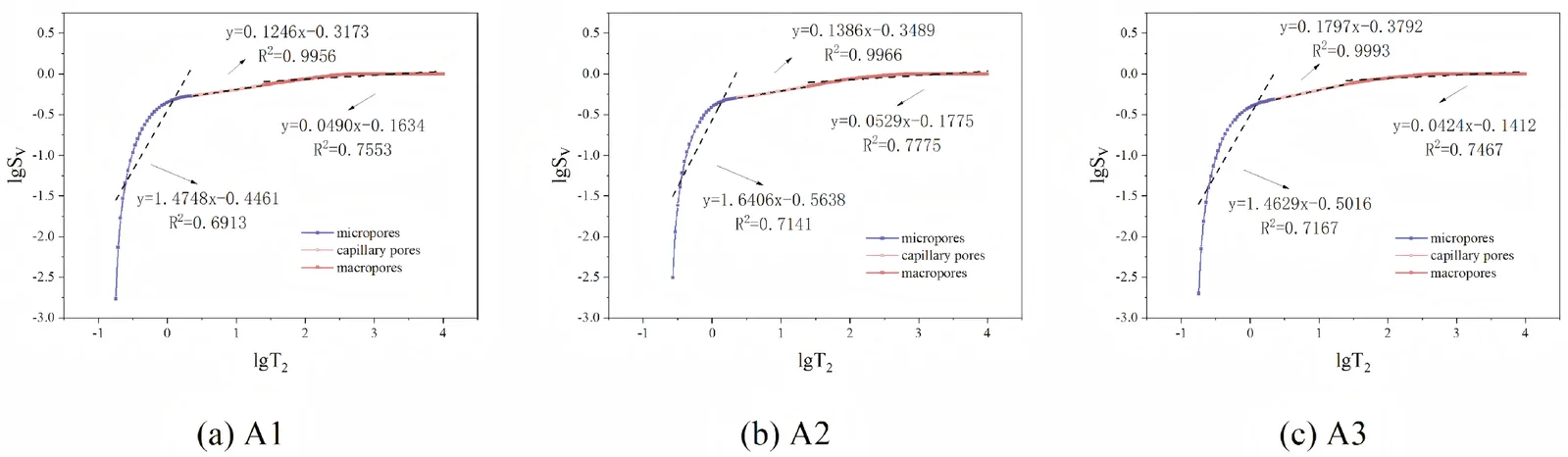
lgT_2_-lgS_v_ curve of concrete with different RHA contents. (a) A1; (b) A2; (c) A3.

Fig 14 and Table 6 present the fractal dimensions of PCM concrete with incorporating 10% to 30% FA, respectively. With the increase in the replacement level of FA, the fractal dimension of capillary pores in concrete with 30% FA added was higher than that with 10% FA, increasing by 0.0528, while the fractal dimension of macropores was lower, decreasing by 0.0107. The incorporation of FA modifies the particle gradation of the cementitious materials, and its lubricating effect reduces the excessive water absorbed by RHA, thus decreasing the number of capillary pores formed by unhydrated cement and increasing the fractal dimension. Conversely, at high FA dosages (e.g., 30%), the reduced cement content may cause a localized increase in the effective water-to-cement ratio. This can result in more residual macropores with a higher degree of connectivity after water evaporation, thereby reducing their overall structural complexity.

**Table 6:** The fractal dimension of concrete with different FA contents.

	D_micro_	K	R^2^	D_capillary_	K	R^2^	D_macro_	K	R^2^
A3	1.5371	4.36%	0.7167	2.8203	2.00%	0.9993	2.9576	−1.07%	0.7467
A6	1.4443	10.13%	0.7025	2.8711	0.24%	0.9700	2.9493	−0.78%	0.7913
A9	1.4763	8.14%	0.6843	2.8731	0.17%	0.9776	2.9469	−0.70%	0.7908

**Fig 14:**
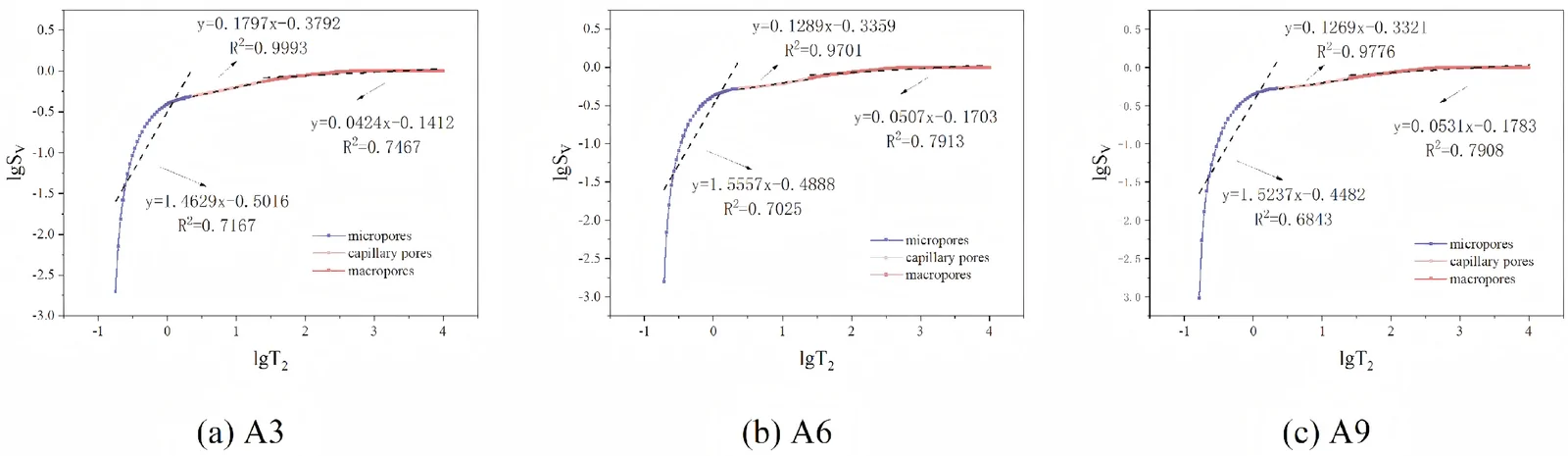
lgT_2_-lgS_v_ curve of concrete with different FA contents. (a) A3; (b) A6; (c) A9.

### 3.7 Correlation analysis

Fig 15 presents a correlation matrix illustrating the relationships between the macroscopic properties and pore characteristics of concrete. The color of the circles is used to distinguish positive and negative correlations—red for positive correlations and blue for negative correlations. The size of each circle represents the strength of the correlation, a larger circle size denotes a stronger correlation.

**Fig 15:**
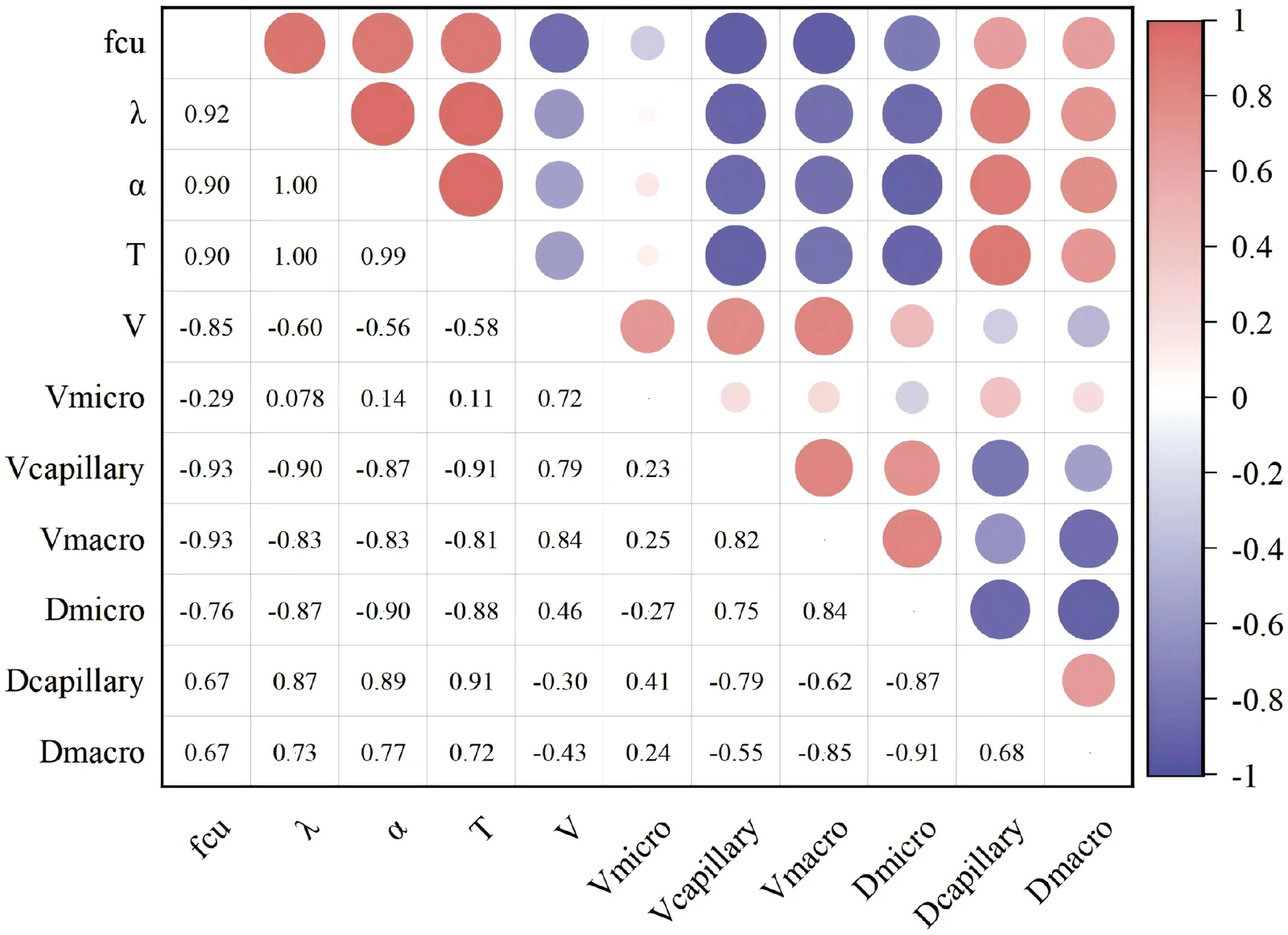
Correlation analysis of macroscopic properties and microscopic indices.

Strength was negatively correlated with pore volume, and the correlation with capillary pore volume was particularly strong, with a correlation coefficient of 0.93. Strength was positively correlated with fractal dimension, meaning a higher fractal dimension corresponds to higher strength. However, the complexity of the pore structure had a smaller impact on strength than pore volume.

The thermal conductivity and diffusivity both were negatively correlated with pore volume and positively correlated with fractal dimension. A particularly strong association was observed with the capillary pores, showing correlation coefficients of 0.90 and 0.87 with their volume and fractal dimension, respectively. This demonstrates that the thermal performance of concrete is governed not only by porosity but also critically by pore structure complexity.

Similarly, the temperature fluctuation amplitude was negatively correlated with pore volume and positively correlated with fractal dimension. In other words, a larger pore volume coupled with a lower fractal dimension resulted in a smaller temperature fluctuation. The positive correlation with fractal dimension is attributed to the incorporation of different materials.

## 4. Conclusion

This study systematically compared the effects of simultaneously incorporating 5%−15% RHA and 10%−30% FA on the properties of PCM concrete, thereby effectively reducing indoor heat loss in winter and lowering the energy consumption required to maintain indoor comfort. The main conclusions are as follows:

(1) The workability of concrete is adversely affected by RHA, evidenced by a 35–42% slump loss at 15% incorporation. Conversely, FA improves workability, capable of increasing slump by up to 55%. Therefore, blending both materials synergistically achieves an acceptable workability level.
(2) When the RHA addition amount is 10%, the concrete strength reaches its peak. However, if the RHA dosage continues to increase, it will hinder the hydration reaction process, leading to a decrease in strength. The optimal addition amount of FA is also 10%. When the amount of FA replacing cement is too large, its positive effect on enhancing strength cannot offset the negative impact brought by cement replacement.
(3) Thermal constant tests indicate that incorporating RHA leads to a significant reduction in thermal conductivity, with measured decreases ranging from 18%−25%, and the thermal diffusivity decreases by 23%−29%. With the incorporation of FA, the changes in the thermal constants are limited. Comparing RHA and FA at the same dosage, RHA is more effective in reducing the thermal constants of concrete.
(4) The thermal response tests reveal that the inclusion of RHA and FA enhances the thermal buffering capacity of concrete, effectively slowing temperature changes. The results of the temperature-time curve are consistent with the thermal constant results. Under an equivalent total replacement level, RHA is more effective than FA in improving the thermal inertia. The peak temperature of PCM concrete decreases by 2.3 °C after incorporating RHA and FA.
(5) After an appropriate amount of RHA and FA is incorporated into PCM concrete, the pore volume decreases. Continuing to increase the RHA content leads to a gradual increase in capillary pores and a decrease in fractal dimension, while the volume of macropores first decreases and then increases, with little change in the fractal dimension of macropores. As the FA content increases, the pore volume, especially the volume of macropores, increases, and the fractal dimension decreases.
(6) Correlation analysis shows that there is a strong relationship between macroscopic properties such as thermal performance and pore characteristics, especially with a high degree of correlation with capillary pore volume and fractal dimension.

## Supporting information

S1 File Data. (ZIP)
